# SNAP: a structure-based neuron morphology reconstruction automatic pruning pipeline

**DOI:** 10.3389/fninf.2023.1174049

**Published:** 2023-06-14

**Authors:** Liya Ding, Xuan Zhao, Shuxia Guo, Yufeng Liu, Lijuan Liu, Yimin Wang, Hanchuan Peng

**Affiliations:** ^1^Institute for Brain and Intelligence, Southeast University, Nanjing, China; ^2^Guangdong Institute of Intelligence Science and Technology, Zhuhai, China

**Keywords:** neuron morphology reconstruction, bioinformatics, image processing, post-processing, dendrite tracing

## Abstract

**Background:**

Neuron morphology analysis is an essential component of neuron cell-type definition. Morphology reconstruction represents a bottleneck in high-throughput morphology analysis workflow, and erroneous extra reconstruction owing to noise and entanglements in dense neuron regions restricts the usability of automated reconstruction results. We propose SNAP, a structure-based neuron morphology reconstruction pruning pipeline, to improve the usability of results by reducing erroneous extra reconstruction and splitting entangled neurons.

**Methods:**

For the four different types of erroneous extra segments in reconstruction (caused by noise in the background, entanglement with dendrites of close-by neurons, entanglement with axons of other neurons, and entanglement within the same neuron), SNAP incorporates specific statistical structure information into rules for erroneous extra segment detection and achieves pruning and multiple dendrite splitting.

**Results:**

Experimental results show that this pipeline accomplishes pruning with satisfactory precision and recall. It also demonstrates good multiple neuron-splitting performance. As an effective tool for post-processing reconstruction, SNAP can facilitate neuron morphology analysis.

## 1. Introduction

Characterization of neuron cell type is an international research frontier in neuron science (Zeng and Sanes, 2017). Neuron morphology is considered to be a critical component of neuron cell type identification (Ascoli et al., 2008). In recent years, there has been considerable development of techniques, including sparse, robust, and consistent fluorescent labeling of a wide range of neuronal types (Peng et al., 2021) and fluorescence micro-optical sectioning tomography (fMOST; Gong et al., 2016). With these techniques, reconstruction of single-neuron morphology from optical microscopy images has become possible and now has an essential role in neuron science. Researchers have developed various manual, semi-automated, and automated neuron reconstruction tools for digital reconstruction of neuron morphology (Meijering, 2010). Research institutions have also held competitions and established worldwide projects, such as the DIADEM competition (Liu, 2011) and BigNeuron (Peng et al., 2015; Manubens-Gil et al., 2023). A large number of automated neuron reconstruction algorithms exist. For example, the 3D Visualization-Assisted Analysis software suite Vaa3D (Peng et al., 2014) has more than 32 plugins, including ENT (Wang et al., 2017), APP (Peng et al., 2011), APP2 (Xiao and Peng, 2013), NeuTube (Zhao et al., 2011), MOST (Wu et al., 2014), and ST (Chen et al., 2015).

Nevertheless, neuron morphology reconstruction remains an unsolved problem (Li S. et al., 2019). The wide variety of brain images in terms of background noise, complicated branching patterns, and clutter of neuron fibers presents challenges for automated neuron reconstruction. Existing automated reconstruction algorithms are generally effective only for a few specific data sets. Owing to the complexity of the images and the limitations of automated reconstruction algorithms, these algorithms are unsuitable for whole-brain images. Moreover, for data sets with a low signal-to-noise ratio and dense neuron distribution with neuron fiber entanglement, the existing reconstruction algorithms do not show satisfactory performance. Pre-processing algorithms, including multi-scale enhancement (Zhou et al., 2015), CaNE (Liang et al., 2017), and filtering-based enhancement (Guo et al., 2022) aim to enhance images by reducing background noise and improving image contrast. Deep learning–based approaches have been investigated for neuron tracing. Among them, weakly supervised learning (Huang et al., 2020) and false negative mining (Liu et al., 2022) are proposed to rescue and connect the weak and broken neurites in the segmentation step for reconstruction; subgraph connection (SGC) method (Huang et al., 2022) starts from prediction map obtained by CNN to link the broken reconstruction; crossover structure separation (CSS) method (Guo et al., 2021) is proposed to detect the crossover structures and generate deformed separated neuronal fibers in the images to eliminate entanglements in reconstruction. However, even with these pre-processing and advanced deep learning–based approaches, the results of automated reconstruction still contain complex errors and cannot be used directly in analysis. To obtain gold-standard morphology reconstruction, researchers need to curate reconstruction results with manual reconstruction platforms such as Vaa3D (Peng et al., 2014), TeraVR (Wang et al., 2019), or FNT (Gao et al., 2022); however, such manual annotation is labor-intensive and time-consuming, limiting the throughput of the morphology reconstruction workflow.

**Figure 1:**
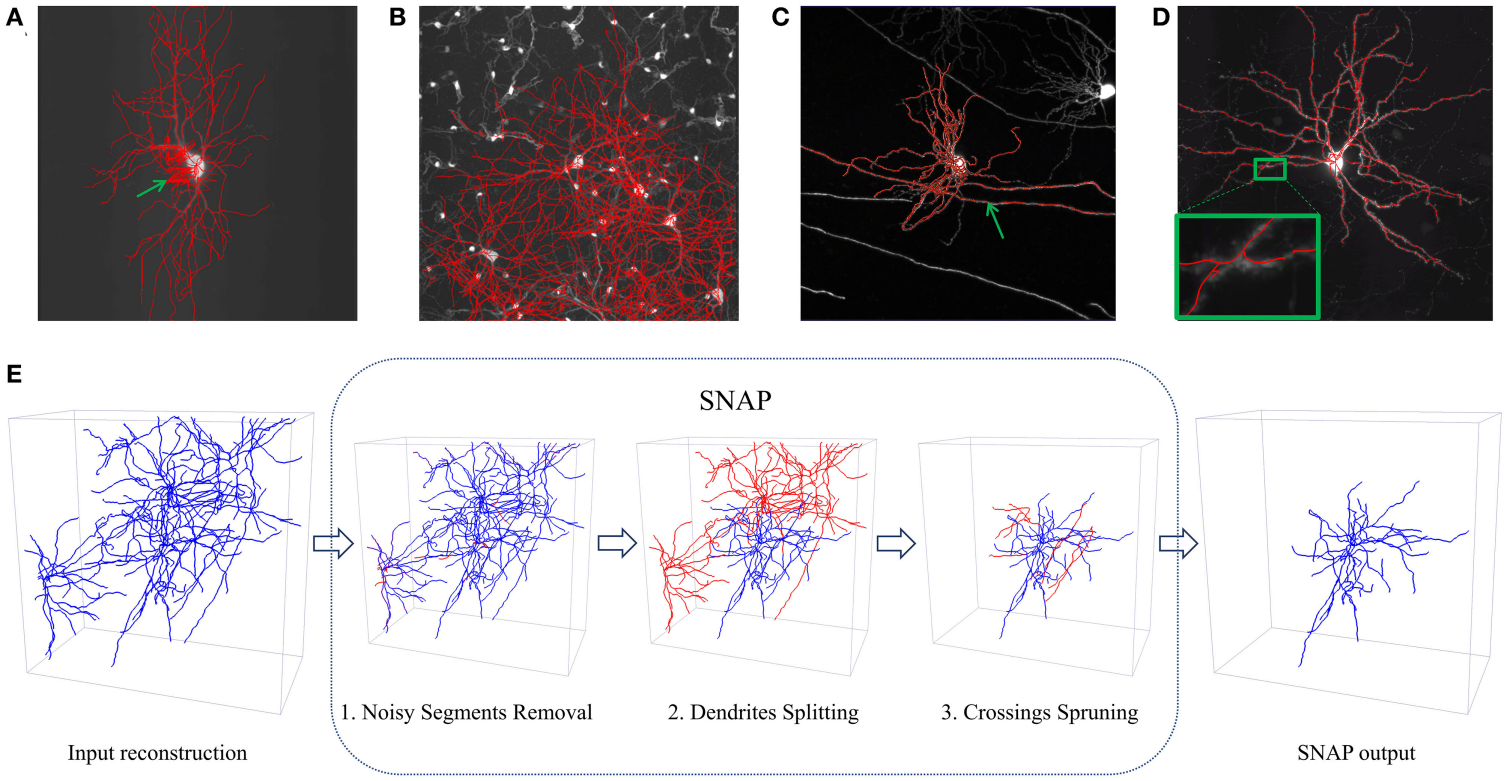
Examples of automated reconstruction results and SNAP pipeline processes. **(A–D)** Display MIP images of neurons overlaid with automated reconstruction results in red. The green arrows point to places of erroneous reconstruction. Four situations of erroneous extra reconstruction segments are shown: **(A)** noisy segments in the APP2 results; **(B)** dense neuron fibers with entanglement with other neurons in the APP2 results; **(C)** entanglement with passing neuron fibers in the ST results; **(D)** entanglement with the neuron itself in the ENT results. **(E)** The workflow of the SNAP pipeline illustrated with an example. In each step, the corresponding type of wrong segments are pruned away. Step 1 prunes noisy segments; step 2 prunes entanglements with other dendrites; step 3 prunes crossings involving passing fibers of other neurons or fibers of the neuron of interest itself. Note that blue segments are the result of each step, while red ones are the pruned–way ones in each step.

In morphology reconstruction systems, therefore, the manual annotation time should be reduced to achieve high throughput, which means the errors resulting from automated reconstruction must be reduced. We closely studied the errors in reconstruction results from several automated algorithms, including ENT (Wang et al., 2017), APP2 (Xiao and Peng, 2013), and ST (Chen et al., 2015). Based on observations of a vast number of samples (see Supplementary material, Section 1), we identified several types of error: missed reconstruction and erroneous extra reconstruction due to entanglement, noise, or other artifacts. Note that by the term “entanglement” in this paper, we mean neuron fibers very close to each other in optical microscopy images that are difficult to distinguish, resulting in “crossing” structures in reconstruction. These intertwined reconstructions within the same neuron or from different neurons constitute significant challenges for automated reconstruction. Figures 1A–D show examples from various situations of automated neuron reconstruction results with errors. With manual annotation for error type on the error sample set (see Supplementary material, Section 1), we found the majority (around 63.53%) were erroneous extra reconstructions (false positive), whereas a reasonable number (around 24.16%) were due to missed reconstruction (false negative), and the rest (around 12.31%) were combined errors. On the other hand, we carried out a survey for the annotation personnel on their opinion on which of the two tasks, annotating automated reconstruction results with some extra segments or reconstruction with some missing segments, would be more time-consuming or tiring. Ninety percentage of the group believed the process of eliminating extra reconstruction segments is more time-consuming or laborious than adding missing segments. Reducing erroneous extra reconstruction segments could expedite the process of manual annotation. Therefore, it is a promising approach to prune automated reconstruction results.

In the literature, there are several papers describing post-processing of automated reconstruction results using various methods, e.g., G-Cut (Li R. et al., 2019), ray-shooting based repairer (Yu et al., 2021), and solemnization algorithm (Jiang et al., 2020). However, only some of these studies focused on the pruning of results. In the challenging scenario of group neuron reconstruction in densely labeled regions with entanglement of dendrites from multiple neuron cells, the main errors are erroneous extra reconstructions due to crossings, as mentioned above. Solutions to this problem include G-Cut (Li R. et al., 2019), NeuroGPS-Tree (Quan et al., 2016; Zhou et al., 2021), and TREES Toolbox (Cuntz et al., 2010), which separate densely intertwined neurons. G-Cut determines which neuron a node belongs to by judging the angle between the local segment and the line connecting the soma and the node. NeuroGPS-Tree identifies spurious links (“bridges”) between the reconstructions of two neurons in an iterative manner and separates the neurons by removing certain ends of bridges. TREES Toolbox employs competitive branch order in neuron splitting. However, most of these software tools do not handle other errors, such as entanglement errors within the same neuron and errors involving other axons passing by, which are essential tasks in pruning.

This paper proposes SNAP, a structure-based neuron reconstruction automated pruning pipeline. It aims to prune away errors in the reconstruction results while keeping correct reconstructions, thereby speeding up further curation. It also separates the entangled reconstructions of multiple neurons as this is part of the pruning problem. We focus particularly on dendrite reconstruction as this is the basic component of neuron reconstruction. The dendrite corresponds to the near-soma region, which serves as the first image block of UltraTracer (Peng et al., 2017) for complete neuron morphology reconstruction. When post-processing in this first block reduces errors, fewer wrong reconstructions will be made when UltraTracer adaptively explores and traces neighboring subareas, which will improve the overall reconstruction performance. When developing SNAP, we thoroughly studied dendrite structure and identified models for the four main categories of errors we needed to prune. SNAP has three main steps, and the pipeline is illustrated in Figure 1E. The algorithms are described in Section 2. The performance of our proposed SNAP pipeline is validated (in Section 3) by applying it to automated reconstruction results and comparing the pruned results with those of gold-standard manual annotation. We demonstrate that a great proportion of erroneous extra reconstruction segments are removed, and thus the reconstruction quality is improved substantially.

## 2. Methods

**Figure 2:**
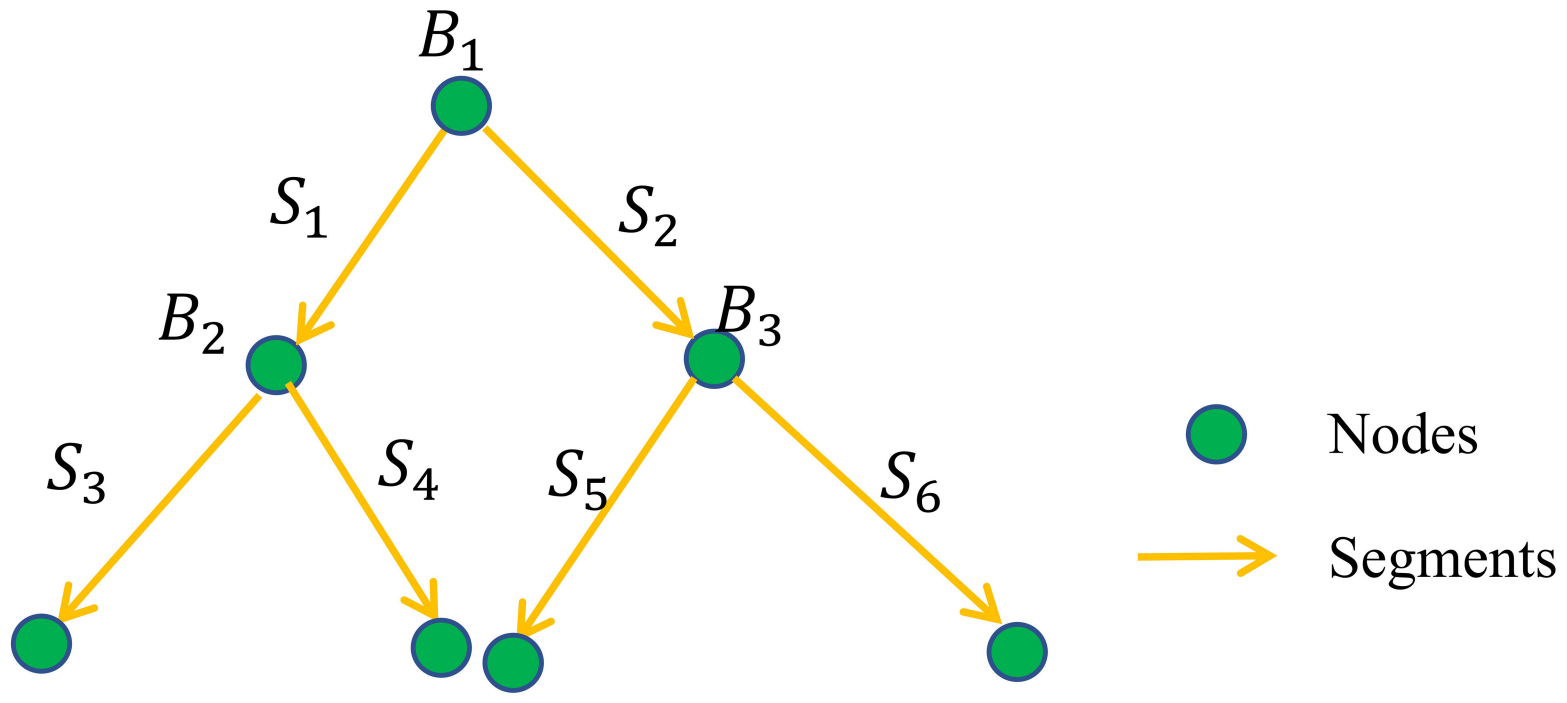
SNAP reconstruction data structure: illustration of converted reconstruction format of directed segments and node-based tree structure.

The digital neuron morphology reconstruction results can be organized into a tree-like set of nodes with parent–child relationships (O'Halloran, 2020) and are usually stored in standardized SWC files (Cannon et al., 1998). In SNAP, the reconstructions are first converted into a segment-based tree data structure, as shown in Figure 2. We denote the segment set as {*S*_*i*_}, *i* = 1, 2, ..., *N*, where *N* is the total number of segments. The parent and child relationships of nodes in SWC format are converted into the parent and child relationships of the segments. The nodes, including the soma point, bifurcation points, and endpoints, have a facilitating role, and we denote the corresponding node set as {*B*_*j*_}, *j* = 1, 2, ...*M*, where *M* is the total number of nodes. If a segment is the furthest segment from the soma, without any child segments, we call it a leaf segment. The level of the segment, $S_{i}^{Lev}$, is calculated as the number of segments *S*_*i*_ that must be passed through to reach a leaf segment. For example, the leaf segment's level is 0, its parent segment's level is 1, and so on. Note that the segments are oriented, in the direction of reconstruction outwards from the soma. SNAP aims to identify the erroneous extra reconstruction segments in the segment set of {*S*_*i*_}.

Note also that SNAP targets single-neuron reconstruction, so if there is more than one connected structure in the reconstruction results, the tree associated with the soma understudy will be kept and worked on, whereas the other parts (including some broken reconstruction fragments) will be discarded.

Four major categories of erroneous extra reconstruction are identified in the statistical analysis mentioned above. *C*_1_ are segments caused by noise in the background. The other three categories are segments caused by entanglement with dendrites of other neurons (*C*_2_), axons of other neurons (*C*_3_), or the same neuron (*C*_4_). The pipeline is designed to deal with all four categories. It starts with the relatively easier category *C*_1_ to simplify the situation and then moves on to the harder cases. Thus, the pipeline deals first with the *C*_1_ type in Step 1, then with *C*_2_ in Step 2, and finally with *C*_3_ and *C*_4_ in Step 3, as shown in Figure 1E.

### 2.1. Step 1: removal of noisy segments (*C*_1_)

**Figure 3:**
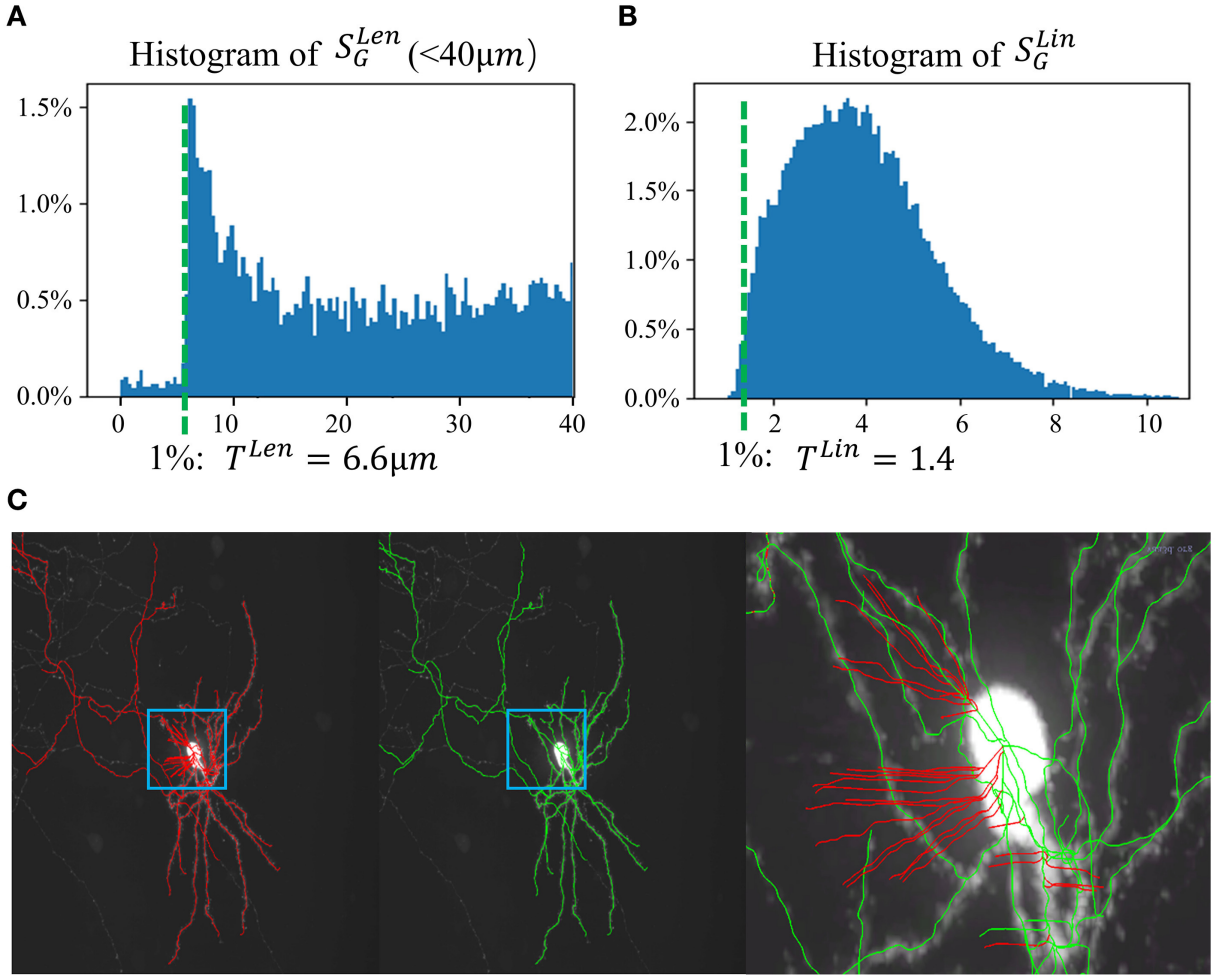
SNAP step 1. **(A)** Histogram of leaf segment lengths from the training set. One percentile of the population is taken as *T*^*Len*^ = 6.6 μ*m*. **(B)** Histogram of leaf segment linearity from the training set. One percentile of the population is taken as *T*^*Lin*^ = 1.4. **(C)** Example result of step 1. From left to right: MIP of a neuron image overlaid with APP2 results shown in red, MIP overlaid with SNAP results shown in green, zoomed-in image of the small region overlaid with SNAP results shown in green, with pruned segments shown in red.

Segments in *C*_1_ are usually caused by noise in the background, including noise due to microscopy imaging, signals from irrelevant particles, or the halo of a strong signal. In general, these noisy segments are leaf segments and are relatively short. A key observation is that the linearity of these segments is weak, whereas the linearity of true neuron fiber segments is strong. Using a set of gold-standard manual annotations, statistical analysis of the length of leaf segments $S_{G}^{Len}$ is performed, as shown in Figure 3A. One percentile of the population is set as a reasonable threshold (*T*^*Len*^) to identify such short segments. Furthermore, the linearity feature of each segment is calculated. The “anisotropy” values of each node in the segments, λ_1_, λ_2_, andλ_3_ (λ_1_ > λ_2_ > λ_3_), are the eigenvalues of the node; hence, the linearity feature is calculated as $S_{i}^{Lin}=\frac{1}{N_{i}}\overset{N_{i}}{\underset{j=1}{\sum }}{\text{λ}}_{1}^{j}/{\text{λ}}_{2}^{j}$, where *N*_*i*_ is the number of the node in *S*_*i*_. Based on the histogram of $S_{G}^{Lin}$ of the training data set, a valid segment usually has a *S*^*Lin*^ value greater than *T*^*Lin*^ (as in Figure 3B), which is one percentile of the population. In applications, leaf segments are removed using rules based on *T*^*Len*^ and *T*^*Lin*^. This process is repeated until no further leaf segments can be removed. Figure 3C shows an example of Step 1.

Removing *C*_1_ is a simple procedure and does not involve much of the neuron structure. Part of the purpose of Step 1 is to avoid artifacts caused by these short and noisy segments from persisting into Steps 2 and 3. Steps 2 and 3, which deal with the remaining wrong segments *C*2, *C*3, and *C*4, are much more closely related to the dendrite structure and represent the main contribution of our proposed pipeline.

### 2.2. Step 2: separation of entangled dendrites (*C*_2_)

For the pruning of segments involving nearby neurons, which usually have their dendrites entangled with the dendrites of the current neuron, we need to define locations to separate the reconstruction into multiple neurons.

**Figure 4:**
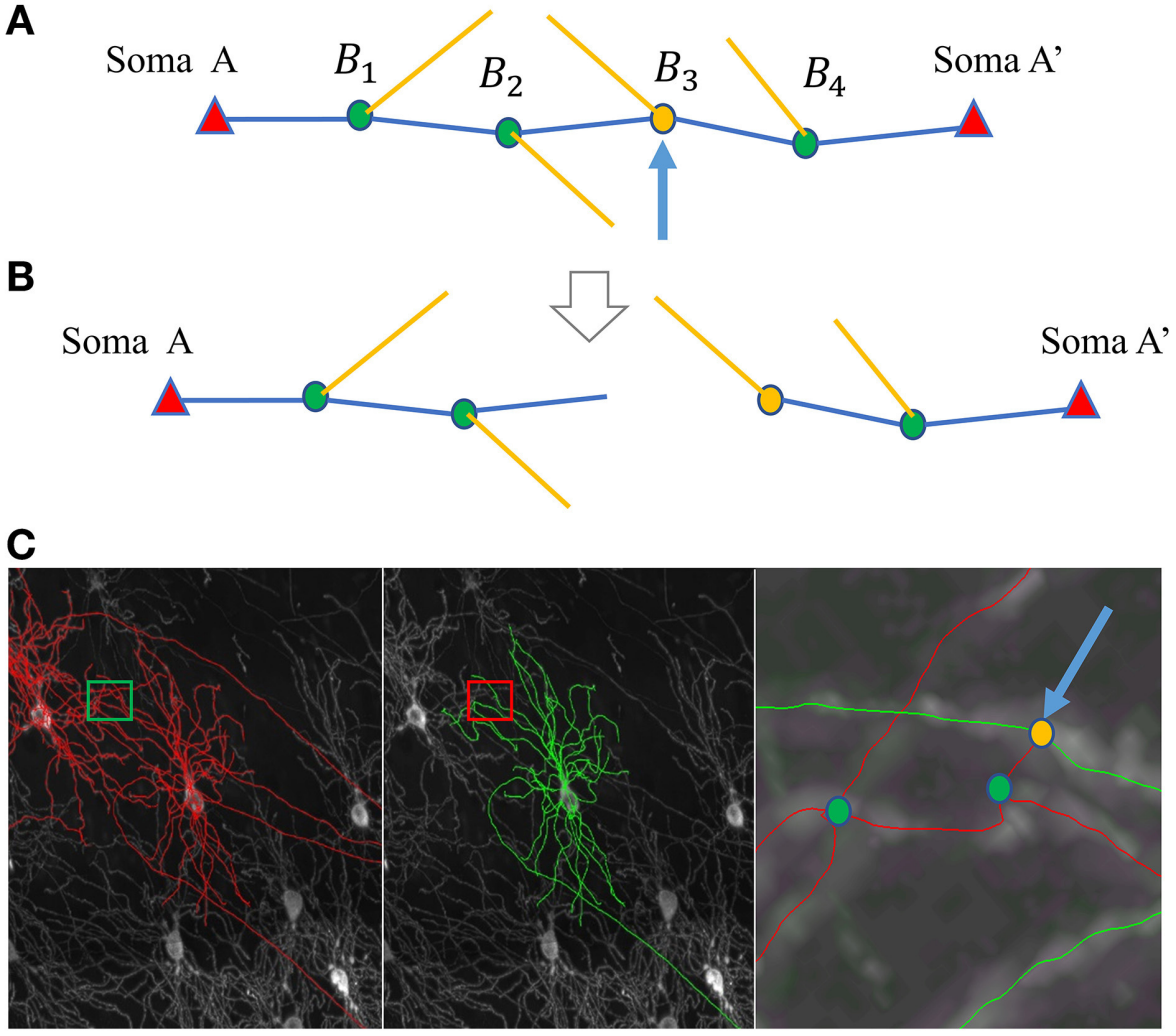
SNAP step 2 for separating neurons. **(A, B)** Illustrate the separating process, where somata, bifurcation points, and segments are represented by triangles, dots, and lines, respectively. **(A)** The original path linking the two somata, A and A'. The blue segments construct the main path, and the yellow segments are the child segments. The blue arrow points to the identified best dividing bifurcation point. **(B)** The resultant divided parts. **(C)** An example of separation. From left to right: MIP of the original image block overlaid with APP2 results shown in red, overlaid with SNAP results shown in green, and a zoomed-in view of the small region around the entanglement.

Without loss of generality, we assume a pair of neurons with soma *A* and soma *A*′ that need to be separated. The path linking *A* and *A*′ has bifurcation point set *B*_*i*_, for *i* = 1, 2, ...*N*_*B*_, where *N*_*B*_ is the total number of the bifurcation points on this path (Figure 4A). Each bifurcation point is a candidate separation site, and we need to identify the bifurcation point that best separates the path. After the separation, there are two reconstructions on the path: *R*_*A*_ for the neuron with soma *A*, and $R_{A^{\prime }}$ for the neuron with soma *A*′ (Figure 4B). Using this bifurcation point to separate the path should be beneficial to the reconstruction of both neurons. The goal of Step 2 is to maximize the sum of the likelihood of branching patterns in *R*_*A*_ and $R_{A}^{\prime }$. Here, weight *W*_*X*_*B*__*i*__ is introduced to reflect the likelihood of *B*_*i*_ belonging to the neuron with soma *X* (where *X* is either *A* or *A*′). The summation of weights for each bifurcation is used to reflect the joint likelihood. Thus, the best bifurcation point for separation, *B*_*s*_, can be identified by:


(1) $$\begin{array}{l} \underset{s}{arg max}W_{s}=\overset{s}{\underset{i=1}{\sum }}W_{AB_{i}}+\overset{N_{B}}{\underset{j=s}{\sum }}W_{A^{\prime }B_{j}}. \end{array}$$

Using statistical analysis of the branching pattern in training data sets, the weight is defined based on the angle of main path segments and child segments and on the distance between bifurcation points and the soma location (details in Supplementary material, Section 2). Putting this weight into the argmax target *W*_*s*_ above, the best separation point *B*_*s*_ can be identified. Then, the two neurons are separated at this point into two reconstructions, *R*_*A*_ and $R_{A^{\prime }}$.

This process is applied to all paths linking neuron pairs; thus, they can all be separated. When applying pruning to the neuron of interest, reconstructions are separated, and the resulting reconstructions belonging to other neurons are removed.

Note that in the process above, the soma locations are known. In addition, most of the abnormally long paths also have the problem of entanglement with other neurons, even when no somata of other neurons are close by. In such cases, a patch is added that uses the endpoints of those paths as “fake” soma locations for purposes of the separation. Figure 4C shows an example of separation.

### 2.3. Step 3: pruning for “crossings” (*C*_3_ and *C*_4_)

Finally, *C*_3_ and *C*_4_ are pruned. In both these categories, the “wrong” segments are caused by local entanglement, involving either passing fibers of other neurons (*C*_3_) or fibers of the neuron of interest itself (*C*_4_). Crossings due to entanglements are commonly found in automated reconstruction results and contribute to the majority of wrong reconstructions that are troublesome to manually correct. The removal of these two types is important and a key target of SNAP.

All branching structures in the reconstruction are checked. Based on the bifurcation number in the local neighborhood of “crossings”, there are two main types of structures: (1) one bifurcation without nearby bifurcations; and (2) more than one bifurcation nearby.

**Figure 5:**
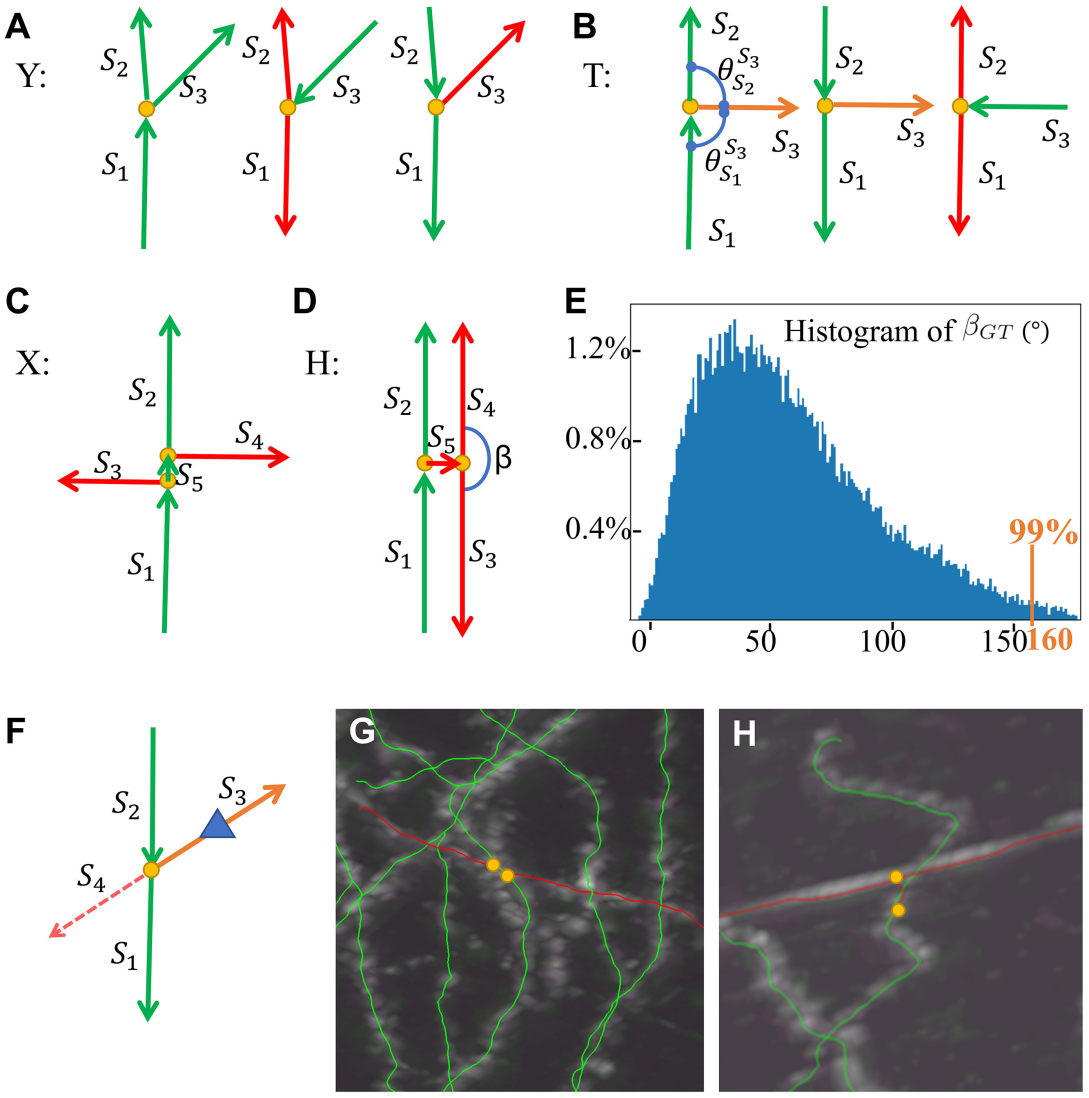
SNAP step 3 models and examples. **(A)** **Y** models. **(B)** **T** models. **(C)** **X** model. **(D)** **H** model. **(E)** Histogram of *β*_*GT*_ in the training data set. **(F)** Reconstruction for suspicious **Y** models as degenerated **X** models. **(G)** An example of a pruned **X** structure. **(H)** An example of a pruned **H** structure. Red arrows for wrong segments, orange arrows for suspicious segments, yellow dots for bifurcation points, and a blue triangle for the reconstruction direction.

One bifurcation structure can be modeled as **Y** or **T**, as in Figures 5A, B. For **Y**, the two segments that are best aligned are termed *S*_1_ and *S*_2_, and the other segment as *S*_3_, and *S*_2_ is assigned to the segment with a smaller angle with *S*_3_. Different situations of parent–child segment relationships are examined. When the parent segment is *S*_1_, we have a typical bifurcation; otherwise, the child segments could represent an error involving other dendrites or axons and thus a wrong segment due to “crossing.” When the parent segment is *S*_3_, *S*_1_, and *S*_2_ are considered wrong and will be removed. When the parent segment is *S*_2_, then *S*_3_ is suspicious; the determination of *S*_3_ will be solved in the degenerated **X** case as described later. When the angle $\theta_{S_{1}}^{S_{3}}$ and $\theta_{S_{2}}^{S_{3}}$ are both close to 90 degrees, the **Y** type becomes a **T** type, which is processed in a similar way to the **Y** type.

The multiple bifurcation types are generally double or more **Y** with bifurcation points very close to each other. We define the confusing types with two bifurcation points as models of **X** and **H** as in Figures 5C, D, based on whether the short segment *S*_5_ linking the two bifurcations is correct or not, where it is correct in **X** and wrong in **H**. The child–child segment angle *β* plays a major part in **H** and **X** pruning. The angle threshold *T*^*β*^ is defined as 99% of the child–child segment angle population in the training data set, as in Figure 5E, to define outliers. The **H** model is prioritized for pruning. We identify the pair of child segments that are both leaf segments in this structure (*S*_3_ and *S*_4_ as in Figure 5D). If the angle *β* between them is larger than *T*^*β*^, these segments and their parent segment are pruned away. This process continues recursively until no further **H** can be identified. Then, for **X** models, we identify the segment linking the two bifurcations; the angles between its child segments and “brother” segments (e.g., *S*_3_ and *S*_4_ as in Figure 5C) are all calculated, and the two segments with the maximum angle, if larger than *T*^*β*^, are pruned away. More details of the **XH** model-based method are described in Supplementary material, Section 3.

In real data, there are many **X** and **H** structures with missing segments. As above, a suspicious **Y** or **T** model can be such a **X** or **H** model with missing segments. When the **Y** cases are considered suspicious, they are treated as degenerated cases of **X**. The pipeline has a local “re-tracing” process to help determine the removal. For a suspicious segment, the node with a distance of *Len*_*R*_ from the bifurcation point is used as the starting point, and the rest of the segment is masked out from the image. FastMarching is run to see whether reconstruction grows out to the two other segments (Figure 5F). If so, the segment is considered correct; if not, we believe it can be attributed to the “crossing” that this segment belongs to and hence this segment is pruned away. Figures 5G, H show examples pruned X and H structures.

Note that when the models have even more missing segments, there will be no bifurcation points left, and the segments become single segments. Therefore, single segments need to be checked if they are degenerated cases of **YTXH** structures. “Inflection” points are identified and pseudo-**X** structures are pruned as described in Supplementary material, Section 4.

## 3. Data set and results

### 3.1. Data set

This study was based on three-dimensional images of single neurons acquired from 28 mouse brains with two-photon fluorescence imaging system fMOST (Gong et al., 2016). In this fMOST dataset, the whole-brain image at the second-highest resolution level (with pixel resolution around 0.6 μ*m* × 0.6 μ*m* × 1 μ*m* in the *x*-*y*-*z* axes) was cropped into image blocks of fixed size (512px × 512px × 256px in the *x*, *y*, and *z* dimensions), each covering the dendritic region of a neuron with the cell body (soma) in the block center. We obtained gold-standard manual annotations from SEU-Allen Joint Center and identified the corresponding dendrite reconstruction results in the cropped images. Six hundred of them were randomly selected as the training data set for the statistical analysis throughout this work. Another 1,000 neurons constituted the testing data set, independent of the training data set. SNAP can be applied to reconstruction results from many different algorithms, e.g., ENT, ST, MST, etc. In our experiments here, the original automated neuron reconstruction results were obtained using the Vaa3D-APP2 platform with adaptive intensity threshold and default parameters for the algorithm. We opted for APP2 since it produces high-quality results on the data set we used.

### 3.2. Qualitative evaluation

**Figure 6:**
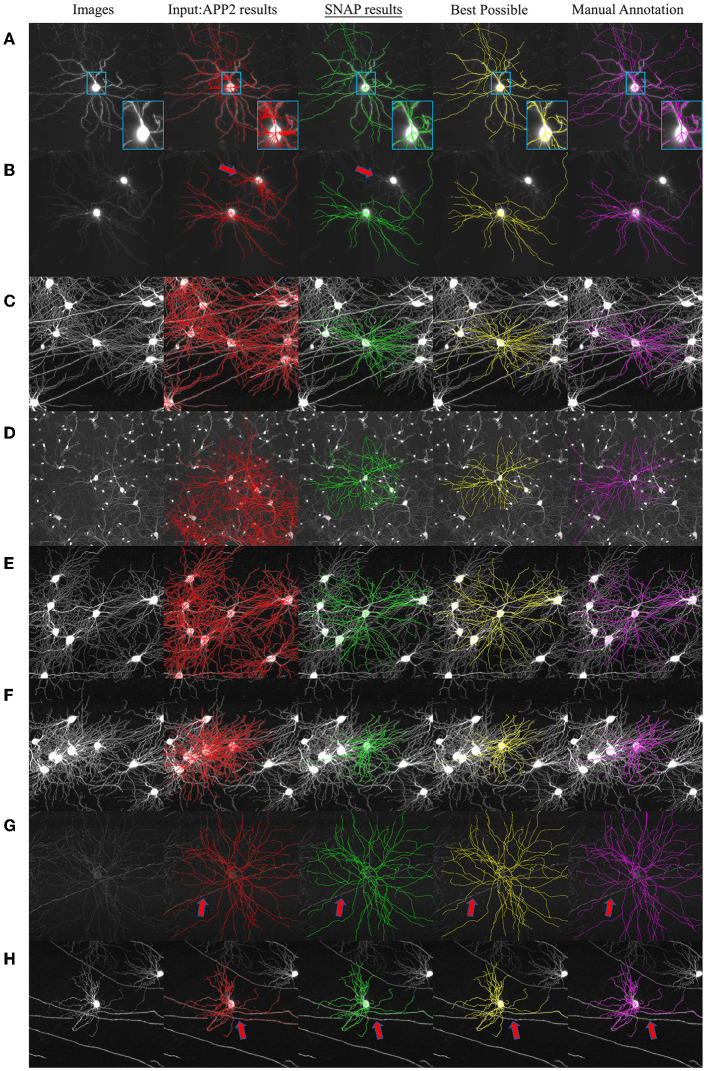
Example SNAP results on fMOST data set. The columns from left to right are as follows: MIP of original image block; overlaid with APP2 results shown in red; overlaid with SNAP results shown in green; overlaid with BP shown in yellow; overlaid with GT shown in magenta. Red arrows point to locations of pruned segments. Examples show pruning results of **(A)** noisy segments (with inset of zoom-in region in light blue boundary boxes); **(B–F)** entanglements with close-by dendrites; **(G)** local entanglement; and **(H)** passing fiber entanglement.

SNAP was applied to the reconstruction results for the 1,000 images in fMOST testing data set. The pruning results were satisfactory. Visual examples are shown in Figure 6. The pruning of *C*1 performed effectively, as exemplified by Figure 6A, which includes zoom-in inset regions highlighting the removal of noisy segments. Multiple-neuron entanglements *C*2 were successfully resolved as in Figures 6B–F, where the reconstruction for single target neurons is separated out from the entangled multi-neuron reconstructions. The pruning of *C*3 & *C*4 entanglement segments was also effective as in Figures 6G, H, where local and passing fiber entanglements were pruned away.

**Figure 7:**
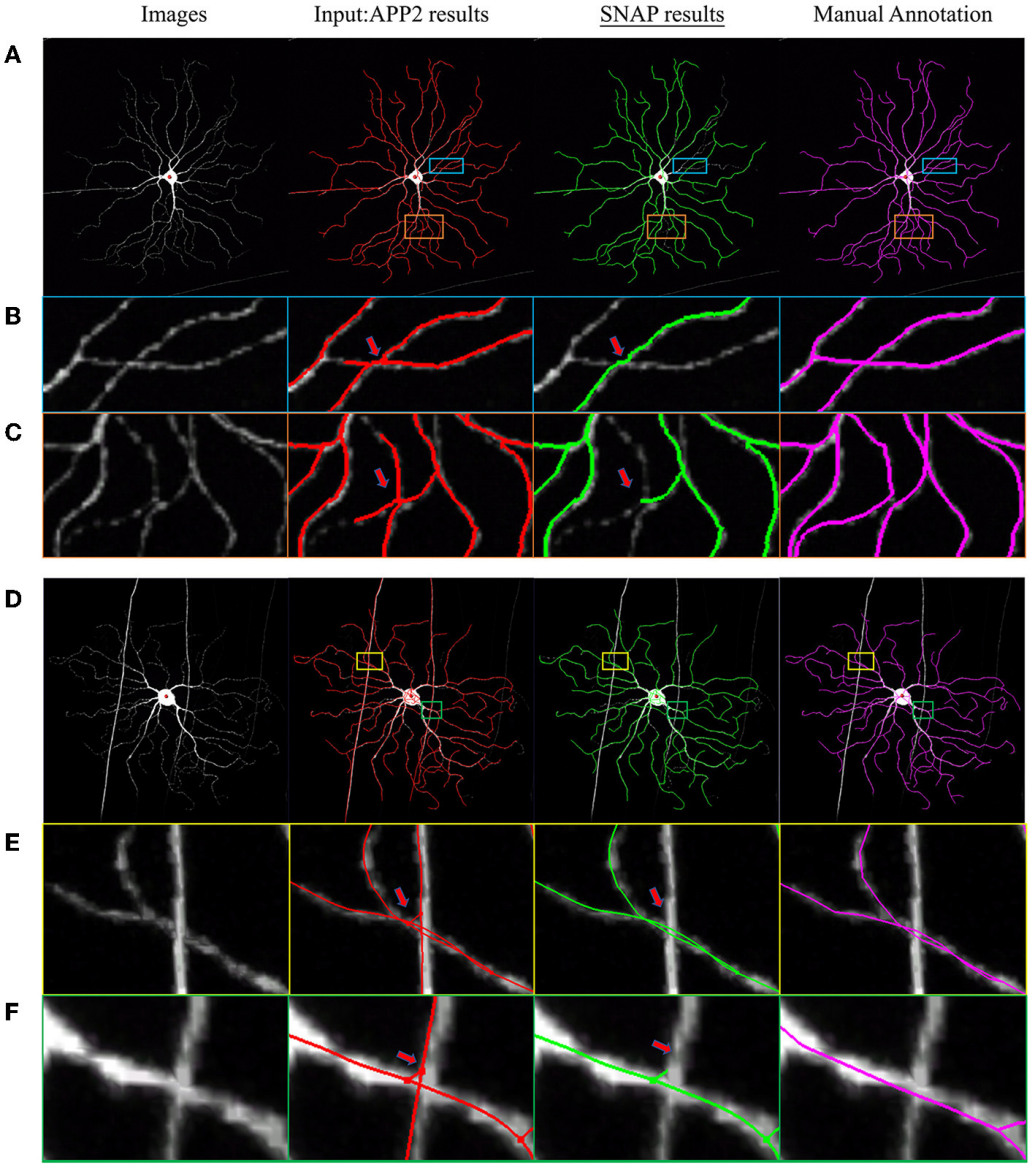
Example SNAP results on BigNeuron data set. The columns from left to right are as follows: MIP of original image block; overlaid with APP2 results shown in red; overlaid with SNAP results shown in green; overlaid with GT shown in magenta. **(A–C)** and **(D–F)** are two sets of examples, where **(A, D)** are full image; **(B, C)** are zoomed-in regions as in the light-blue and orange bounding boxes overlaid on **(A)**, displaying pruning of wrong crossings within the same neuron; **(E, F)** are zoomed-in regions as in the yellow and green bounding boxes overlaid on **(B)** (with slightly different viewing angle), displaying pruning of entanglements with passing fibers.

In order to test SNAP's capability to automatically prune reconstruction obtained by a variety of algorithms from images other than the fMOST dataset above, we checked into BigNeuron (Manubens-Gil et al., 2023), which contains various neuron images with benchmarking reconstruction. SNAP with default parameters was applied to the high-rank automated reconstructions of mouse neuron images. Two examples are shown in Figure 7. In the first example as in Figure 7A, the input automated reconstruction was obtained with 3D Tubular Models (Santamaría-Pang et al., 2015), and pruning of wrong crossings within the same neuron was successful (see the zoom-in regions in Figures 7B, C). In the second example, as in Figure 7D, the input automated reconstruction result was obtained with NeuroGPS-Tree and pruning of entanglements with passing fibers was effective (see the zoom-in regions in Figures 7E, F).

### 3.3. Quantitative performance evaluation

To demonstrate the performance of SNAP, we provide a quantitative evaluation. As gold-standard manual annotation results were available, we could compare the output to this “ground truth” (GT) to determine the accuracy. However, since we start with the automated reconstruction results, and the algorithm prunes but does not add any missing segments, a direct comparison is not an appropriate choice. Hence, the “best possible pruned” result (BP) is calculated by removing all the segments from APP2 results that are not present in the GT based on their distance to GT segments (see Supplementary material, Section 5 for the BP calculation). As BP keeps some short segments due to noise that are very close to ground truth segments, we evaluate step 1 separately and then evaluate steps 2 and 3 (without the involvement of the short segments in step 1).

In the first experiment, the performance of step 1 (pruning C1 segments) was checked. One hundred dendrites were randomly chosen from the testing data. The original reconstruction and pruned results were presented to human annotators, who were asked to label the correctly pruned segments and also the wrongly pruned ones. The results showed that out of the 48, 793 segments, $N_{total}^{S1}=10,400$ segments were pruned away, of which $N_{TP}^{S1}=10,233$ were true noisy segments, and $N_{FP}^{S1}=165$ were correct segments that were mistakenly removed. In the analog to a detection problem (where a true positive corresponds to correctly pruned segments), SNAP step 1 was quantitatively evaluated as follows.


$$\begin{array}{l} \text{Precision of step 1: }PPV^{S1}=\frac{N_{TP}^{S1}}{N_{TP}^{S1}+N_{FP}^{S1}}=98.4\%.\\ \text{False discovery rate of step 1: }FDR^{S1}=\frac{N_{FP}^{S1}}{N_{TP}^{S1}+N_{FP}^{S1}}=1.6\%. \end{array}$$

Note that we did not ask annotators to determine false negatives, owing to the heavy manual labor cost of this task. Hence, no sensitivity or miss rate is given here. However, such segments would go through later steps and possibly be included in the evaluation of steps 2 and 3.

Having validated step 1, we used the current pruned results to calculate the BP results. Examples of the BP results and also the GT are shown in Figure 6. BP was not identical to GT, since BP results are the biggest matching subset of APP2 results. Note that although there has been research involving further post-processing to rescue missing segments, this is beyond the scope of this paper.

In the second experiment, steps 2 and 3 were evaluated together. The SNAP results are compared with the “ground truth” given by BP. For the 1,000 neurons in the testing data set, there were $N_{total}^{S23}=398,846$ segments, and $N_{P}^{S23}=248,378$ segments were removed, of which $N_{TP}^{S23}=238,537$ were true wrong segments and $N_{FP}^{S23}=9,841$ were mistakenly removed correct segments. There were also $N_{FN}^{S23}=58,132$ segments that should have been pruned but were not. In the analog to a detection problem, SNAP steps 2 and 3 were quantitatively evaluated as follows.


$$\begin{array}{l} \text{Precision of steps 2 and 3: }PPV^{S23}=\frac{N_{TP}^{S23}}{N_{TP}^{S23}+N_{FP}^{S23}}=96.0\%. \end{array}$$

$$\begin{array}{l} \text{Sensitivity of steps 2 and 3: }TPR^{S23}=\frac{N_{TP}^{S23}}{N_{TP}^{S23}+N_{FN}^{S23}}=80.4\%. \end{array}$$

To reflect the differences in length among segments, we further evaluated SNAP steps 2 and 3 using segment length. Altogether, $L_{total}^{S23}=36273284.5$ pixels; we removed $L_{P}^{S23}=22429289.5$ pixels, where $L_{TP}^{S23}=1262893.0$ pixels were true positives, $L_{FP}^{S23}=1166393.8$ pixels were false positives, and $L_{FN}^{S23}=4393361.0$ pixels were false negatives. The evaluation above could be re-done as $PPV_{L}^{S23}=94.8\%$ and $TPR_{L}^{S23}=82.9\%$.

### 3.4. Comparisons with other approaches

To fully evaluate the proposed algorithm, we compared the performance of SNAP with that of other approaches. G-Cut (Li R. et al., 2019), NeuroGPS-Tree (Quan et al., 2016), and TREES toolbox (Cuntz et al., 2010) are post-processing algorithms that can deal with the dissembling of multiple neuron entanglement by “separating” the neuron reconstruction results. From a single-cell perspective, these methods also prune away wrong segments that do not belong to the cell of interest. Hence we evaluate the pruning performance of these software tools and compare them. To ensure a fair comparison of pruning performance, we would like to rule out effects from different automated reconstruction methods. So the same input reconstruction should be provided to them. Here APP2 reconstruction results were used as the base reconstruction results for all of these tools.

Of the 1,000 testing neurons, 598 involved multiple-neuron involved. The four tools were applied to all these samples with given soma locations and used to quantitatively evaluate each result for the neuron of interest. Specifically, when there were several dendrites close to the neuron of interest, the result was the separated and processed reconstruction of this neuron, disregarding the results for other neurons; this evaluation was done for 454 neurons (samples not included are: ones with multiple neuron, but APP2 results don't involve entanglements with multiple neurons; ones with no pruning happened thus precision is not defined). Three commonly used metrics, precision, sensitivity, and F1-score, were calculated for each neuron. For this specific separation problem, we adopted Miss-Extra-Score (MES; Xie et al., 2011) as used in the evaluation of G-Cut (Li R. et al., 2019), as MES provides a global view for neuron reconstruction based on accuracy and undesired components. MES was originally defined as $\frac{\left(S_{G}-S_{miss}\right)}{\left(S_{G}+S_{extra}\right)}$, where *S*_*G*_ is the total length of all segments in the GT trace, and *S*_*miss*_ and *S*_*extra*_ are the total lengths of missing and extra segments in the automated trace, respectively (compared with the GT). In our pruning setting, MES was reformulated as $MES=\frac{\left(TN+FP\right)-FP}{\left(TN+FP\right)+FN}$. Both segment-based and length-based metrics are presented.

**Figure 8:**
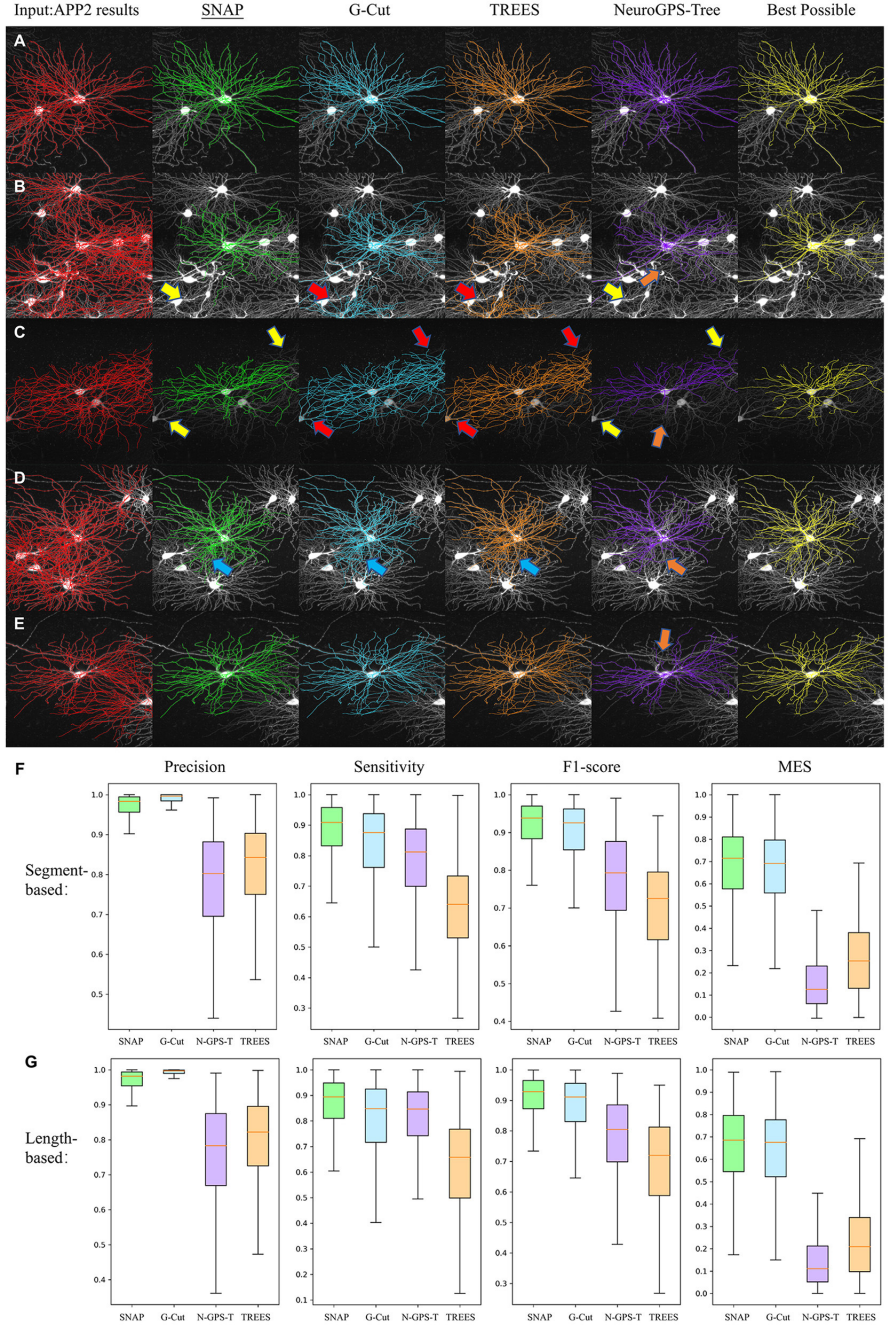
Performance comparison. **(A–E)** Visual examples. From left to right: the original image (displayed as MIP, same for the other sub-figures) overlaid with input APP2 results (red), SNAP (green), G-Cut (blue), TREES Toolbox (orange), NeuroGPS-Tree (purple), and BP results (yellow). The arrows point to locations of some differences between the algorithms: yellow arrows for correct removal, red arrows for unsuccessful removal, orange arrows for over-pruning, and blue arrows for under-pruning. **(F)** The four quantitative metrics based on segments for the four algorithms are shown with box plots. **(G)** Length-based metrics. In **(F, G)**, “N-GPS-T” is used as the abbreviation for “NeuroGPS Tree.”

Figures 8A–E show several visual examples of results. All four algorithms performed reasonably well in separating the target neuron from entangled reconstructions. Some detailed differences are: (1) SNAP and NeuroGPS-Tree are both capable of removing entanglement segments of close-by neurons even when their soma locations are not within the image region. G-Cut and TREES Toolbox rely on the clear definition of all nearby soma locations(as in Figures 8B, C with yellow arrows pointing to the correct removal of these segments in SNAP and NeuroGPS-Tree and red arrows pointing to unsuccessful removal in G-Cut and TREES Toolbox). (2) Similar to (1), SNAP and NeuroGPS-Tree could be on the strict side in pruning(see in Figures 8C–E with orange arrows pointing to over-pruning). (3) In some cases, SNAP, G-cut, and TREES Toolbox have difficulty removing segments in conjunction region of two neurons (see in Figure 8D with blue arrows pointing to under-pruning).

Figures 8F, G show box plots of precision, sensitivity, F1-score, and MES for SNAP, G-Cut, NeuroGPS-Tree and TREES Toolbox. We can see G-Cut has best precision, and SNAP has the best sensitivity, F1-score, and MES scores. Since SNAP and G-Cut perform relatively comparable, we further counted how often SNAP or G-Cut algorithms performed better than the other for each neuron, and how often they performed equally well, based on these four metrics (Table 1). Overall, SNAP had relatively lower precision but better sensitivity, F1-score, and MES; hence, in general, SNAP outperformed the rest of the algorithms.

**Table 1:** Tables of performance comparison results for SNAP and G-Cut.

**Segment-based**			
**Metric**	**SNAP is better (%)**	**SNAP = G-Cut (%)**	**G-Cut is better (%)**
Precision	33	19	**48**
Sensitivity	**60**	2	39
F1-score	**55**	1	44
MES	**47**	11	42
**Length-based**			
**Metric**	**SNAP is better (%)**	**SNAP = G-Cut (%)**	**G-Cut is better (%)**
Precision	38	8	**54**
Sensitivity	**61**	1	38
F1-score	**54**	0	46
MES	**45**	11	**45**

Both segment-based metrics (top) and length-based metrics (bottom) are presented. The bold numbers indicate the superior algorithm in the comparison.

**Figure 9:**
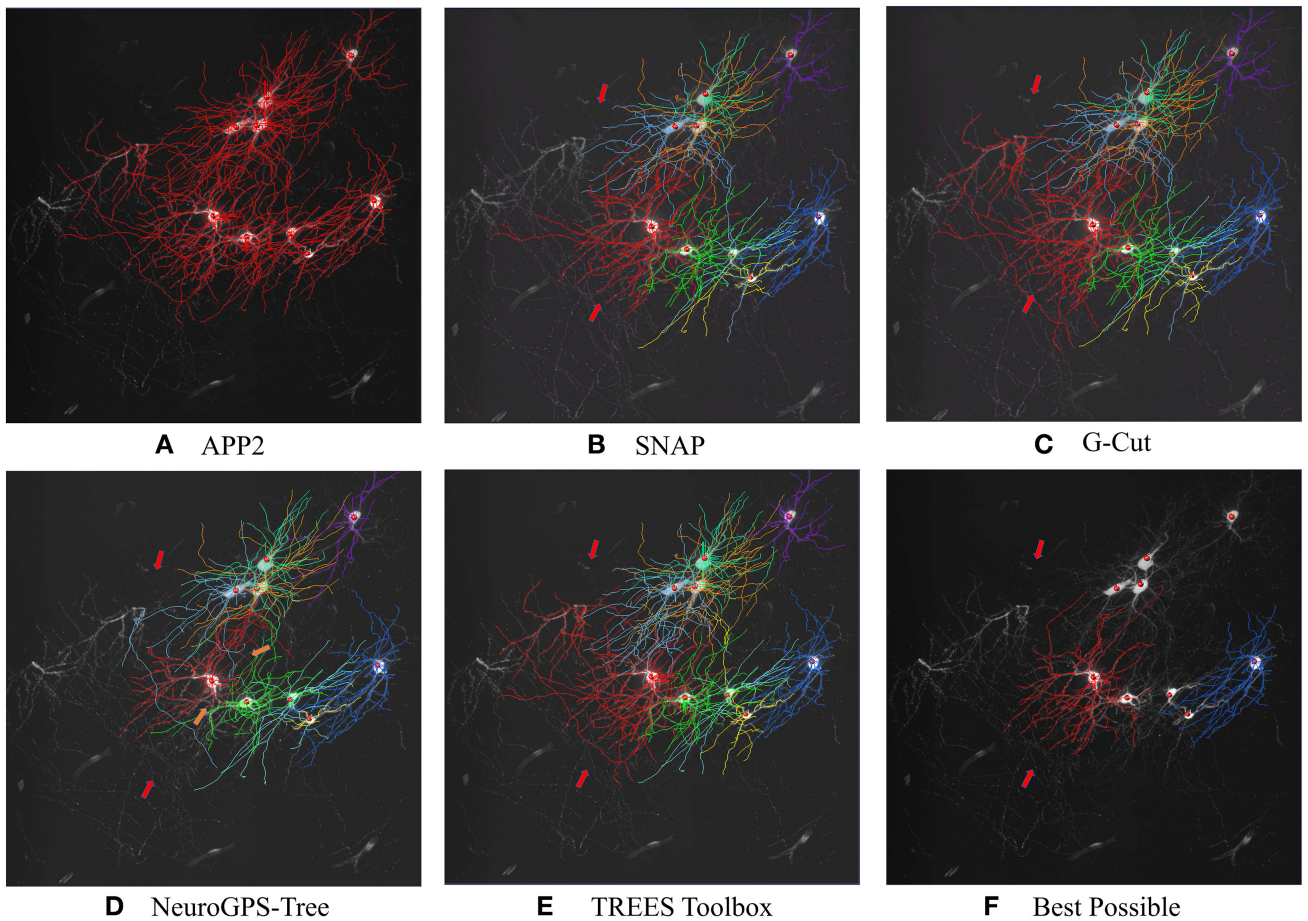
A multiple neurons separating and pruning example. MIPs are displayed and overlaid with reconstruction. Different neurons are shown in different colors that are consistent across the results of all algorithms and manual annotation. **(A)** APP2 results as input. **(B)** SNAP results. **(C)** G-Cut results. **(D)** NeuroGPS-Tree results. **(E)** TREES Toolbox results. **(F)** Best Possible results of the two neurons with manual annotation. The red arrows point to locations with entanglements between neurons which some algorithms can remove and some cannot. Orange arrows point to locations of over-pruning.

With the ability of separating target neuron from entangled reconstruction, SNAP natural achieved multiple neuron separation functionality in dense and entangled neuron reconstruction problems by pruning w.r.t. each of the neuron. One example was shown for its multiple neuron separation performance and compare it with that of G-Cut, NeuroGPS-Tree, and TREES toolbox. An image block with nine neurons (mostly dendrite portions) was reconstructed with APP2. As shown in Figure 9, all nine neurons were entangled as one reconstruction. We applied the four algorithms in separating the nine neurons with soma locations given. Figures 9B–E show the SNAP results, the G-Cut results, the NeuroGPS-Tree results, and the TREES Toolbox. We can see that all algorithms could separate the neurons reasonably well. There are some differences within these results, and similar to examples in Figure 8 there are some over pruning and under pruning involved along the separation. Figure 9F provides the Best Possible pruned results from APP2 results with manual annotation of the two neurons visible in this field of view (the rest of the neurons don't have manual annotations). From the visual comparison, we can see SNAP achieved good separation and pruning for this group of neurons.

## 4. Software availability

This study was conducted with the support of the Vaa3D platform (v.3.601). The released binary and the source code for the Vaa3D platform are available through the GitHub release page of vaa3d.org (https://github.com/Vaa3D). The software implementation of the method presented here was developed in C++ and built as a plugin in the Vaa3D framework (Peng et al., 2014) with Qt-4.7.2 installed. SNAP implementation was tested using both CentOS and Windows operating systems. It is available for download at https://github.com/Vaa3D/vaa3d_tools/tree/master/hackathon/XuanZhao/SNAP. Guidance for use of the plugin is included in the README.txt file.

## 5. Conclusion and discussion

In this paper, we present SNAP, a structure-based neuron morphology reconstruction automated pruning pipeline. It incorporates statistical analysis and structure modeling into rules for removing erroneous extra segments, thereby improving neuron reconstruction workflow throughput. Experimental results, especially for quantitative evaluation with high precision and recall, demonstrate the effectiveness of SNAP. SNAP also achieved neuron separation in entangled neuron problems.

Note that the methods in SNAP depend on statistical priors and use empirical values as thresholds. Here, it is important to point out that the prior knowledge drawn from careful study of gold-standard manual annotation data is on the different types of errors and structural models, which are independent of the choice of the automated reconstruction algorithm. SNAP can be applied to the results of any automated reconstruction algorithm.

As SNAP reduces the number of wrong segments, manual curation can be speeded up. The results obtained with SNAP could serve as an improved basis for further post-processing algorithms, e.g., repair algorithms to make up the missing branches. SNAP could also be applied to manual annotation as a QC tool to identify segments that are possibly wrong. Hence, it is a powerful tool facilitating high-through neuron morphology reconstruction.

## Data availability statement

The original contributions presented in the study are included in the article/Supplementary material, further inquiries can be directed to the corresponding authors.

## Author contributions

LD conceptualized the project, developed the algorithm with help from the team, and wrote the manuscript. XZ assisted with algorithm development and implemented the software. YL contributed to algorithm development. LL led the annotation of the gold-standard data. SG and YW contributed to algorithm development and manuscript writing. HP supervised the project. All authors contributed to the article and approved the submitted version.
