# Supplementary material for: SNAP: a structure-based neuron morphology reconstruction automatic pruning pipeline
Source: Front Neuroinform. 2023 Jun 14;17:1174049. doi: 10.3389/fninf.2023.1174049 (PMC10303825; doi:10.3389/fninf.2023.1174049)
Supplement: Supplementary file 1 [file Data_Sheet_1.pdf]

Supplementary Material

1 RECONSTRUCTION ERROR STATISTICS

To discover the statistical distribution of errors in automated reconstruction results, we built an error sample set. A group of neurons in an fMOST brain image with ID 18454 were traced automatically. The results were compared with the corresponding manual annotations. Based on the distance from reconstruction to manual annotation, 10088 error samples were identified. From this error set, several types of error are observed, including missed reconstruction, erroneous extra reconstruction due to entanglement, noise, or other artifacts, and others. To get a statistical analysis of this error set, human annotation was carried out. For each error sample, the MIP of the cropped image overlaid with the automated reconstruction and the MIP overlaid with the manual annotation were presented to the human annotators to judge the error type among a list of types, including false positive due to dense signals, false positive and negative due to dense signal, false positive due to noisy background, false positive due to false tip, false negative due to weak signal, and inaccurate reconstruction due boundary issues or other artifacts, etc. Excluding the errors due to inaccurate reconstruction(2232 samples), the rest of the set(7856 samples) involves false positives and/or false negatives, among which 4991 samples are false positives(63.53%), 1898 samples are false negatives (24.16%) and 967 samples contain both false positive and negatives(12.31%). In the subset of false positive samples, 3892 error samples are due to dense signal(77.98% within this subset), 851 error samples are due to noise(17.05% within this subset), 75 error samples are due to false tip(1.50% within this subset) and 173 error samples are due to other artifacts(3.47% within this subset).

2 WEIGHTS IN SEPARATING NEURON RECONSTRUCTION

In SNAP step 2, entangled neuron reconstructions are separated. The weight W_{XB_i} is an estimate of the likelihood of point B_i belonging to the neuron with soma X, which is critical in the design of optimization-based separating point identification.

To design a good weight W_{XB_i}, the branching pattern in dendrites is examined closely. In general, neuron fibers grow along with the directions of the main branches. Specifically, it is more likely that child segments grow in a direction similar to their parent segments but not exactly the same direction. When the automatic reconstruction algorithm mistakenly traced neurons onto other neurons, the child and parent segments are very often not aligned. To get support from the data, statistics are drawn from the training data-set of 600 dendrites(as in Sec.3). The angle α^{CP} of child segments and the extended parent segments form an extended Poison distribution. And the majority of the angles (94.2% of them) are acute (less than 90 degrees). When grouping α^{CP} into 18 bins between 0 to 180 degrees, we denote α^{CP}'s bin index as Φ and fit its distribution (Fig.S1(b)) into a Poisson distribution with $\lambda = 2$.

Here, for the branches along the path linking two somata, the child-parent relationships studied are the branching child segment and its parent segment on the main path. Note that for the same bifurcation point the Φ_{AB_i} and $\Phi_{A'B_i}$ are different since the child-parent relationships are different in the two reconstructions R_A and R'_A. The weight design considers two aspects: the angle α^{CP}, and also the distance between B_i and the soma location, assuming the closer B_i is to the soma point, the higher the weight is. The formula is

as follows:

$$W_{Xi} = W^{\Phi}_{XB_i} \times W^{L}_{XB_i} = \frac{\lambda^{(\Phi_{XB_i})} \exp\left(\Phi_{XB_i}\right)}{\Phi_{XB_i}!} \times \frac{L_{All} - L_{XB_i}}{L_{All}}$$

where L_{XB_i} is the path length from the bifurcation point B_i to soma X, L_{All} is the total length between two somata.

3 MORE DETAILS FOR PRUNING WITH X AND H STRUCTURE

In step 2, C3&4 are pruned. The more than one bifurcations types are complicated. Step 1 helps to make this case slightly simpler. As in Fig.S2(a), a valid such model is basically double or more $\mathbf{Y}$ with bifurcation points very close to each other. The typical $\mathbf{X}$ models as in Fig.S2(b) and $\mathbf{H}$ models as in Fig.S2(c) are described in the main text Sec.2.3, and how to prune them is also presented.

Visually, it seems that both $\mathbf{H}$ and $\mathbf{X}$ are easy to identify; however, in real data, there are many cases with missing segments. A suspicious $\mathbf{Y}$or $\mathbf{T}$ model can be treated as degenerated $\mathbf{X}$ or $\mathbf{H}$ model with missing segments. Further degenerated cases are described in Sec.S4.

In other situations, special cases of $\mathbf{X}$ can be tri-furcations as in Fig.S2(d); there are also $\mathbf{Y}+\mathbf{X}$ cases(with more than 2 bifurcation points) in Fig.S2(e). In these cases, a similar process for $\mathbf{X}$ is applied until all $\mathbf{X}$ models are solved.

4 PRUNING FOR C3&4 WITH "INFLECTION" MODEL

When the $\mathbf{X}$ model has even more missing segments, we found that there are no bifurcation points in that situation, and it becomes one single segment. However, we still need to consider if a single segment could be the degenerated case for an erroneous "crossing" structure. For this purpose, we look for whether there is an "inflection" point to decide whether they are degenerated cross structures and pruning accordingly.

Given a long segment, the initial orientation of the segment is calculated by the first part with a length of Len_R. For each of the nodes on this segment, calculate the orientation based on its local neighbor segment part with Len_R. The angle between these two orientations is checked; when the angle is larger than the threshold θ_I, we consider there is an "inflection" point in this segment, and the node with the largest angle is chosen as the "inflection" point.

For each "inflection" point, a quick procedure similar to the "retracing" is carried out to determine whether this inflection point should be pruned(Fig.S2(f)). The node in the "outward" direct with a distance of Len_R is used as the starting point; while the further outwards nodes are masked out, FastMarching is run to see if tracing is going to the original branch. If so, the "inflection" is due to a true turning structure of neuron fiber. If not, it is caused by degenerated "crossing" structure, and so the segment part from the "inflection" point and on is pruned away.

5 OBTAINING BEST POSSIBLE PRUNED RESULTS FOR QUANTITATIVE EVALUATION

For reconstruction results from automated algorithms, we need to identify what will the "Best Possible" pruned result be when given the gold-standard annotation (seen as GT) result. We do this segment by segment. Since an absolute overlap of a segment from the automated algorithm and its corresponding segment in GT is usually impossible, the distance of a segment to GT is studied. This distance D_i^{GT}

for S_i to GT is calculated as: get the distance of each node n_i^j of the segment S_i to GT, from the set remove the largest and the smallest value and then obtain the average. In the case of reconstruction obtained by APP2, the distribution of D_{APP2}^{GT} is shown in Fig. S3, which shows a mixture of a one-side Gaussian with zero mean and minimal standard deviation and a Gaussian with high mean and high standard deviation. The former corresponds to the part of correctly traced segments, and the latter corresponds to the wrong part, which is far away from the GT. We fit this distribution to $0.22\mathcal{N}^t + 0.78\mathcal{N}^f$ with true part $\mathcal{N}^t$ = half-normal of $\mathcal{N}(0, \sigma_t = 1.65)$ and false part $\mathcal{N}^f = \mathcal{N}(\mu_f = 118, \sigma_f = 69)$. Note that the false part is pretty far away from the true part. We define a segment to be "present" in GT by having its average node distance to GT smaller than T_D^{BP}, where T_D^{BP} is defined as $3\sigma_t \approx 5$. With this rule screening through all segments, BP is obtained.

6 SUPPLEMENTARY TABLES AND FIGURES

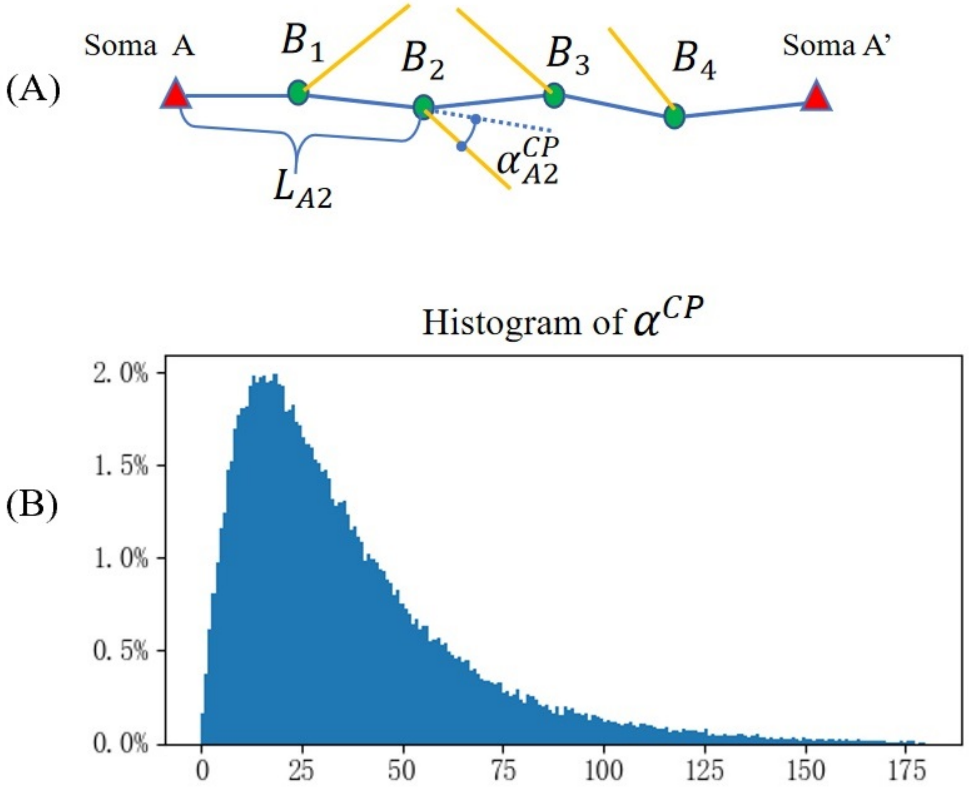

Figure S1. SNAP step 2 model for separating close-by neurons. (A) Model of the path connecting two neuron cell bodies with α_{A2}^{CP} and L_{A2} labeled. (B) Distribution of angle α^{CP} between parent–child segments from the training dataset.

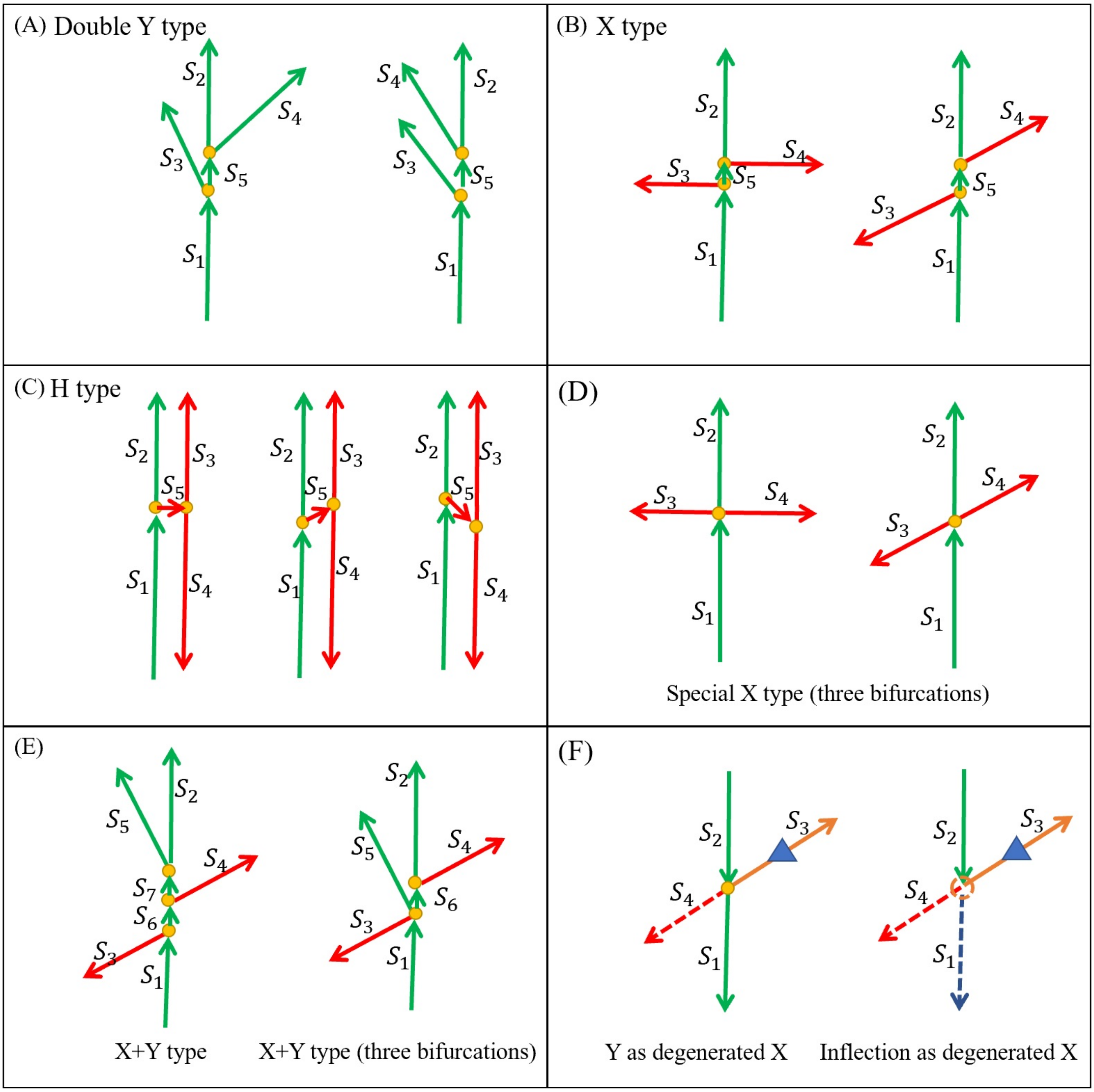

Figure S2. SNAP step 3 **X** and **H** models, special cases and examples. (A) Double **Y** models. (B) **X** models. (C) **H** model. (D) Special **X** models. (E) **X+Y** models. (F) Retracing for suspicious **Y** models as degenerated **X** models and inflection models using retracing. Legends: Red arrows for wrong segments, orange arrows for suspicious segments, yellow dots for bifurcations points, and blue triangles for retracing directions.

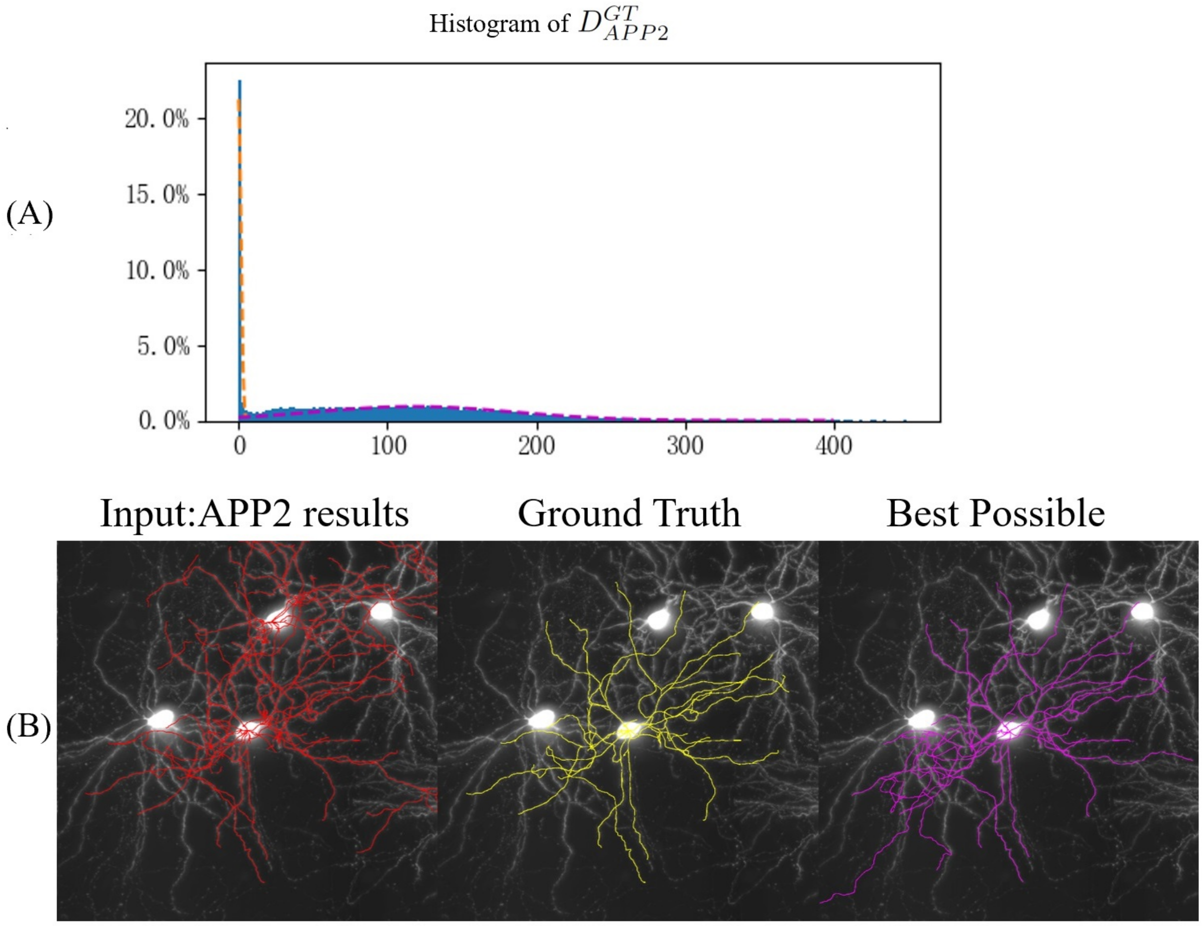

Figure S3. Best Possible pruned results acquisition. (A) Distribution of D^{GT}_{APP2} with fitted models of one-sided Gaussian $\mathcal{N}(0, \sigma_t = 1.65)$ shown in orange and Gaussian $\mathcal{N}(\mu_f = 118, \sigma_f = 69)$ shown in magenta. (B) Example image overlaid with reconstructions of the input reconstructions of APP2 (red), GT (yellow), and BP (magenta) results.
